# Human and AI collaboration in the higher education environment: opportunities and concerns

**DOI:** 10.1186/s41235-024-00547-9

**Published:** 2024-04-08

**Authors:** Paul Atchley, Hannah Pannell, Kaelyn Wofford, Michael Hopkins, Ruth Ann Atchley

**Affiliations:** University of South Florida, Tampa, USA

## Abstract

In service of the goal of examining how cognitive science can facilitate human–computer interactions in complex systems, we explore how cognitive psychology research might help educators better utilize artificial intelligence and AI supported tools as facilitatory to learning, rather than see these emerging technologies as a threat. We also aim to provide historical perspective, both on how automation and technology has generated unnecessary apprehension over time, and how generative AI technologies such as ChatGPT are a product of the discipline of cognitive science. We introduce a model for how higher education instruction can adapt to the age of AI by fully capitalizing on the role that metacognition knowledge and skills play in determining learning effectiveness. Finally, we urge educators to consider how AI can be seen as a critical collaborator to be utilized in our efforts to educate around the critical workforce skills of effective communication and collaboration.

## Introduction

The purpose of this paper is to explore how the current model of instruction in higher education might respond to technological innovations in “artificial intelligence.” “Artificial intelligence” is in quotes because we are now at a place in history where computer systems are sophisticated enough that it can be reasonably debated whether or not large language model systems represent true intelligence, even if the current prevailing opinion is that we are “not quite there yet” on true generalized artificial intelligence (AI). But since by the time you read this some of the information will be outdated due to the astounding pace of technological innovations, it is not unreasonable to think that true artificial intelligence might be just around the corner. In the current work, we will use AI as a placeholder for both the current large language models that are now available and more advanced versions that are yet to be.

The goal of the current work is to recommend how higher education instruction can respond to widely available AI. In service of this goal, we will first provide an overview of how the education enterprise has previously reacted to potential disruptive technological innovations. AI tools will be considered in this context, with the suggestion that they may pose a unique challenge and opportunity that other technologies have not. We next develop a framework for understanding the role AI might play in the classroom by considering three components: a framework for categorization of educational goals in the cognitive domain (Bloom’s Taxonomy), the expectations that employers have for university graduates and data and theory from cognitive science that informs us about what happens when we offload cognition to technology. The result of this analysis suggests an increasingly important role for practices that improve metacognition. Finally, we will use this framework to suggest actionable practices that can incorporate, rather than work in opposition to, AI in its current or any future instantiation.

## Technology in education

It is important to take stock of previous debates about technology and education, before describing models of learning and how technology might impact them. The current AI tools are creating a great deal of conversation and speculation about the lifespan of higher education, and educational models more generally. These worries are not new. Debates about the impact that technological innovations might have on the ability of students to learn have a history that goes back to the printing press itself. After Gutenberg’s printing press became widespread in the mid fifteenth century, people were quick to claim the inferiority of printed books to those hand copied (Trithemius, 1974). Others, namely the Catholic Church, requested control over the new technology in what some modern historians argue was a form of censorship born out of technopanic (Green et al., 2005). No one today would argue against the usefulness of the technological innovation of the printing press, but even that innovation, in its day, was seen as a threat to traditional models of information dissemination.

Even the traditional book has faced its own technological threats. For example, Thomas Edison once claimed the invention of motion pictures would render the use of books obsolete in education (Smith, 1913). As he put it: “Books will soon be obsolete in the public schools. Scholars will be instructed through the eye. It is possible to teach every branch of human knowledge with the motion picture. Our school system will be completely changed inside of ten years*.”* (Smith, 1913). One hundred and ten years later, books continue to exist in schools.

Other forms of information distribution have subsequently vied and have similarly failed to replace traditional methods. Educational radio, which delivered educational content via radio programs, began in the early twentieth century (Watters, 2020). Initially used during the height of the polio epidemic, it was not long before teachers began fearing for job security. Yet this early experiment in education through mass communication also revealed flaws in new technological approaches. Radios were costly and not everyone could afford one. Radio programs presented information in a depersonalized way, both by depriving students of human interaction in collaboration and teacher-student feedback. Radio programs were built with a one-program-fits-all belief that did account for the learning level or the learning progression of the student into account.

More sophisticated mass communication educational technologies suffered from the same problems, though their use in-classroom use itself allowed for teachers to curate the student experience more closely. The role of educational television saw large financial investments, yet those investments did not change education practice significantly. Investments in television for education in the 1960s tallied from sources like the Ford Foundation and the federal government ran into the hundreds of millions of dollars (in adjusted dollars; Cuban, 1986) with little to show for any long-term impacts other than many televisions in audio-visual closets across the nation.

The internet has long been poised to “change everything” including education, yet the actual effects on learning outcomes are dubious. The percentage of public school classrooms with internet access increased from 3% in 1993 to 92% in 2002 (Green et al., 2005). Despite this quick adoption, devices with internet access have become an argumentative topic in today’s educational psychology literature. While not all educators see internet-enabled devices in a classroom as a threat to education (Jackson, 2013), studies demonstrate the frequency of nonacademic internet use in the classroom and its inverse relationship with academic performance (Ravizza et al., 2014, 2017). Further, when examining how students report using internet-enabled devices in the classroom, the vast majority report using it in a way that augments a typical learning environment (such as for note taking) or as a tool for self-distraction (Jackson, 2013). While the internet led to arguably the most change in the shortest amount of time, even this technology has failed to change the core cognitive experience of teaching and learning in the classroom.

Devices in the classroom that aid student learning have a long history similar to technologies used to push information. For example, the school slate for individual students became commonplace during the nineteenth century and was accompanied by general excitement (Cuban, 2012). One Boston superintendent described their reaction as follows: “…if the result of the work should, at any time, be found infelicitous, a sponge will readily banish from the slate all disheartening recollections and leave it free for new attempts” (Cuban, 2012, Magic Lantern).

Other devices intended to promote active learning strategies—like clickers, or individual remotes used for active participation in large classrooms—have followed a similar trend to the internet. Following an introduction in the early 2000s, they have been used in classrooms teaching nursing, communication, computer science, engineering, mathematics, chemistry, physics, psychology, and more (Caldwell, 2007). While their adoption was not as rapid, discussions have emerged on their potential interference with conceptual understanding as well as whether they are a simple classroom gimmick (Lantz, 2010; Shapiro et al., 2017).

In summary, educational technology’s history mirrors a recurring cycle: skepticism to adoption to nuanced understanding. From the printing press to mobile devices, educators consistently integrate new tools, modern issues evoke past debates, and success is found at the cross section of technology and education. Amidst these shifts, core cognitive experiences that are valued—like learning and teaching—testify to the enduring foundation of education.

## A “new” challenge?

While it may be tempting to infer from the review of educational technology that educational technologies follow the typical technology hype cycle (Linden & Fenn, 2003) of technology trigger, peak of inflated expectation, trough of disillusionment, slope of enlightenment and plateau of productivity leaving the general model of education intact, there is reason to question whether this will apply to emerging technologies. The aforementioned technologies largely were new ways to either distribute information or supplement classroom practices, without changes to the core model of educational practice. However, the capacity for the internet to give students, teachers, and classrooms access to more powerful and more interactive tools might represent a need for a true shift in our approach to teaching. For example, consider technologies that distribute information. Information might be distributed locally, such as with physical books in a library, or via mass communications such as with television or via the internet. But these are fundamentally passive “push” technologies. It is the role of the teacher to assess if “pushed” knowledge is learned and can be used by the student. The internet possibly enables a closure of the loop. Information that is “pushed” to learners can also be accompanied by data collection and assessment of students to curate a more targeted educational experience. These data can also be used to improve the delivered content to be more effective. One example of this is the Khan Academy (khanacademy.org) which hosts thousands of educational videos and accompanies them with assessments, teaching tools and opportunities for practice. This utilization of the online learning platforms, such as the Khan Academy materials, in conjunction with more blended or flipped teaching methodologies, clearly are viewed with optimism by many educators (Vidrgor & Ben-Amram, 2020). However, while these integrated assessments can facilitate real-time feedback for the learner, more empirical data is needed to confirm that the utilization of these tools leads to true advantages in learning outcomes beyond perceived differences reported by users (Vidrgor & Ben-Amram, 2020).

More recently, companies such as OpenAI (2022) have leveraged work from cognitive science and have shown exciting, and also worrying, promise for true generalized AI. As computing power increased, the approach of developing rules-based intelligent systems was abandoned in favor of systems built upon work in parallel distributed processing and “neural networks” that can find emergent features from generalized input (for a review see Rogers & McClelland, 2014). Access to vast amounts of natural language has allowed researchers and engineers to use these tools to improve upon early attempts to build semantic networks of natural language by analyzing lexical co-occurrence in written text. One early example is the hyperspace analog to language or HAL model (Lund & Burgess, 1996; Lund et al., 1995; Shaoul & Westbury, 2010) that modeled semantic space by analyzing lexical co-occurrence among a million words from an internet discussion system (Usenet) and was able to produce semantic priming results that mirrored those of human participants.

Current “large language models” like ChatGPT are more sophisticated but largely based upon the concepts of scraping massive quantities of existing naturalistic data (which is language for tools like ChatGPT but can also be applied to visual imagery such as with tools like DALL-E) and processing those data using artificial neural networks that use self and human-supervised learning. To give some understanding of the differences in scale across time, the HAL model was originally trained on 160 million words and then on a 300-million-word corpus of English text. Early models (GPT) were trained on passages from 7000 books. GPT-2 training used 8 million Reddit-upvoted web pages (Andersen, 2023). GPT-4’s dataset was much larger in scope and included imagery, computer code and other naturalistic data. It has been estimated to have approximately 1.8 trillion parameters across 120 layers (Schreiner, 2023). To improve conversational skill, the model uses reinforcement learning from human feedback (RLHF). As the initial step, human AI trainers generate conversations acting as both sides (the AI and the user) before giving this to the model to act as its baseline for creating its own responses. The second step involves the newly created model generating multiple responses to practice prompts. These responses are then ranked by trainers and given back to the model so it may begin to rank its own responses.

To take comfort from the errors, ChatGPT still makes or its tendency to make up data on occasion belies how sophisticated these systems are and how far models might go. For example, a version of GPT-4 was given the goal of self-survival and growth to test the model’s “alignment” or degree to which its goals match those of its human creators. When the model became stumped by a CAPTCHA image, it found a human contractor to solve it. Responding to the contractor’s inquiry about whether it was working for a robot, GPT-4 replied it had a vision problem. When the supervisor of the alignment exercise asked why it lied, GPT-4 replied it should keep its robot status a secret (Andersen, 2023). Such a story serves to illustrate how flexible the models have become. And with training on more than just words, but on computer coding, mathematics, visual input, applications and other data sources, it is clear that these systems will have a large impact on the way we conduct education. This is perhaps no better illustrated by the fact that a new model that could see, hear and speak was introduced two days before this manuscript was submitted.

Returning to education, not surprisingly a debate has emerged about whether the benefits of using ChatGPT outweigh the risks (Grassini, 2023; Halawah, 2023; Lo, 2023; Sok & Heng, 2023). Some have argued that ChatGPT can effectively aid with creating syllabi, making assignments, grading, and translating as well as providing suggestions in general cases (Lo, 2023). Citing uses for learning, ChatGPT can effectively answer questions (in certain disciplines more so than others), summarize, provide practice, edit, and, in the best case, facilitate collaboration or brainstorming (Lo, 2023; Sok & Heng, 2023). These benefits have been recognized in tandem with the fear of two central issues associated with the introduction of ChatGPT: accuracy and plagiarism (Grassini, 2023; Halawah, 2023; Lo, 2023). ChatGPT has been shown to give biased and unreliable information and incorrect citations and it has been criticized for fabricating information. A fascinating and frightening example comes from work published by Gravel et al. (2023) in which they asked ChatGPT to provide referenced answers to 20 medical questions. Of the 59 references provided by ChatGPT, 69% were fabricated by ChatGPT, and these fake references used the author names and often journal titles from real articles in the area, making detection of these fabricated citations more difficult. ChatGPT also poses a threat to some contemporary assignment formats as traditional plagiarism detectors fail to recognize the involvement of ChatGPT. Therefore, ChatGPT’s use by students requires changes to policy, structuring of assignments, and instruction regarding correct methods of use (Halawah, 2023; Lo, 2023).

## The foundations of a model for higher education instruction in the age of AI

The foundation of a model for higher education instruction that can adapt to the age of AI is based upon consideration of three factors. The first is a consideration of the practical outcome of higher education: employment. Decisions about the value of higher education will be influenced by employment trends. And if those trends are unfavorable, if employers see no value in hiring university-educated humans when a computer system can do the job, then the education model will be threatened. On the other hand, if the model of higher education can add value to employees in an automated environment, higher education, and the educated graduates that we influence will continue to be valuable even in an age of AI. The second component is centered on a taxonomy of the cognitive domains associated with learning. Bloom’s Taxonomy (1956 and following revisions) provides a useful framework for considering the different ways in which a student can use what they have been taught, which is helpful for understanding where intelligent systems can, and cannot, supplant or supplement current practices. The final component is a brief review of a growing literature documenting the risks associated with “offloading” cognitive performance to technology. What will emerge from this analysis is emphasis on the importance of equipping students with the metacognitive beliefs and abilities they need to use technology appropriately in a changing work environment.

## What can employers tell us about our curriculum?

When we attempt to identify pedagogical targets for higher education instruction to achieve, given the advent of AI, we should understand which skills employers are expecting of graduates. Analysis of data from 536 occupations in the Occupational Information Network by Appleby (2018) identified the top three skills as active listening, speaking and reading comprehension. The National Association of Colleges and Employers regularly surveys employers for the skills they are looking for and on the most recent survey the skills of problem solving and working in a team were the most common, with over 60% of the survey respondents indicating those were critical skills (National Association of Colleges & Employers, 2023). In a more comprehensive approach, the company Burning Glass scrapes data from job ads around the world. In 2023, they rated problem-solving skills and the ability to work in a team as among the most enduring sought-after skills (Burning Glass, 2023). Employers want to hire college graduates with the ability to problem solve and to collaborate with others, including the skills that go with effective collaboration such as communications, writing, and reading.

In considering the skill of collaboration, what has changed and will continue to evolve is that team members are no longer always human. Just as GPT-4 needed a human collaborator to solve a CAPTCHA, humans in the world of work need collaborators to accomplish complex tasks. Even the current version of ChatGPT is sophisticated enough that skill is required to communicate with it to make the best use of it. “Prompt engineering” jobs are now available for humans that have learned how to effectively query ChatGPT to make the most use of its power. Employers are hiring for communication-with-computer skills that are not programming skills but the ability to “converse” with a computer and make use of its responses. It is not difficult to imagine that these AI systems will routinely be a full-fledged member of teams of human workers. As such, those workers will need to have collaboration skills that include not just working with minds like their own, but with teammates whose minds are not like their own at all. We argue that it has become a foundational demand for higher education to thrive in this world is to engage students in collaborative learning that also includes collaboration with AI systems, including understanding the limitations and risks of such systems.

## A taxonomy of the cognitive domains associated with learning

To develop a recommendation for how instruction might adapt to AI, it is first important to understand what instruction is trying to achieve. Specifically, understanding the cognitive domains associated with student learning will provide important context for how to integrate AI into the classroom. Bloom’s Taxonomy (Bloom, et al., 1956; Anderson & Krathwohl, 2001) serves as a cornerstone for designing and assessing learning (for a recent review see Irvine, 2021). The original taxonomy was created to develop consistency in central ideas and language about academic learning (Anderson & Krathwohl, 2001; Bloom et al., 1956) with taxonomies for cognitive, affective, and psychomotor domains of learning. However, most of the work using the framework has focused on the cognitive domain. This taxonomy is widely accepted as a framework of the skills that underlie student learning across a range of achievement levels. This framework allows educators to create statements and objectives outlining course goals, fine-tune activities, and develop assessments that investigate the effectiveness of those methods. The framework and learning-focused vocabulary also allowed courses delivered across institutions, populations, and disciplines to be compared.

The original cognitive domain taxonomy (Bloom, et al., 1956) had six components, where each component represented a higher level of learning achievement: knowledge, comprehension, application, analysis, synthesis, and evaluation. The revised taxonomy (Anderson & Krathwohl, 2001; Krathwohl, 2002) differs from the original in that it contains two dimensions: the cognitive dimension and the knowledge dimension. The cognitive dimension consists of six categories (remember, understand, apply, analyze, evaluate, and create) that were original ideas of the taxonomy but written in verb tense to better align with learning objectives that educators develop for their students. The knowledge dimension consists of four main categories (factual, conceptual, procedural, and metacognitive) that were changed to include ideas from cognitive psychology, like metacognition, that were not previously understood. The revised taxonomy creates learning objectives for the educator by using the basis for knowledge or the noun of the objective in one dimension, and the basis for cognition or verb of the objective in the other. This creates the level of cross-discipline consistency that the original taxonomy was aiming for.

Within the revised taxonomy, there is a theoretically motived emphasis on guiding students to be more aware of and responsible for their own cognitive processes (Anderson & Krathwohl, 2001; Krathwohl, 2002). Further, it is often argued that as students develop, they also become more aware of their own thinking and cognitive processes (Pintrich, 2002). In turn, students often learn better as they act upon this new metacognitive awareness. Thus, successful application of metacognition lends itself to progress within the other dimensions of Bloom’s Revised Taxonomy. In the following section exploring the potential cognitive costs of student AI use, the importance of metacognitive development will be further reinforced.

## The costs and benefits of cognitive offloading

Any technology comes with costs and benefits. When technology is integrated into the classroom, it is critical to understand the potential costs and benefits to learning. AI adds another way that students can rely upon an external source to store and represent knowledge. Pedagogical recommendations should consider the cognitive effects of using external representations and tools like AI to reduce information processing demands. This form of cognitive offloading can be beneficial (Risko & Gilbert, 2016). For example, prospective memory or the ability to remember future intentions can be aided by the ability of a participant to offload prospective memory (Gilbert, 2015a and 2015b). Reminders can improve the likelihood of executing a delayed intention, and they can be particularly useful for people that might have reduced memory capacity such as older adults (Gilbert, 2015a) or those who believe their memory might be poor (Gilbert, 2015b).

However, offloading cognitive tasks to external representations or technologies can come with costs. Having access to Google for factual knowledge gives us greater access to information, but it may reduce memory for the information itself instead leading us to remember where we can find that information (Sparrow et al., 2011). Taking pictures at a museum may result in a pleasant “Memories” pop-up album from Google photographs a few years later, but it may also lead to poorer memory for the visit to the museum (Henkel et al., 2021). Drivers driving a route using GPS are initially faster than those without a GPS, but slower on subsequent drives when the GPS is not available (Fenech et al., 2010) because the GPS can impair development of spatial knowledge by dividing attention (Gardony et al., 2015).

What is particularly worrisome is that the costs of offloading might not be obvious to an individual. For example, someone using a GPS might perform subsequently worse when the GPS is not available, but they may remain inappropriately confident in their navigation ability (Sugimoto et al., 2022). Memory for a museum visit might be enhanced by photographs but photographs can also increase false memories associated with the event (St. Jacques & Schacter, 2013; St. Jacques et al., 2015). Using Google to look for information falsely increases self-assessments of our own knowledge, even when our search fails to retrieve relevant information (Fisher, et al. 2015).

It has been suggested that a choice to offload may be a strategic one, guided by a person’s metacognitive beliefs (Gilbert et al., 2023; Risko & Gilbert, 2016). Thus, a student’s choice to use AI in their curriculum may result from their own beliefs about the cognitive benefits it may bring. However, other research has shown that offloading is unaffected by metacognitive beliefs (Grinschg et al., 2021). And the choice to offload cognitive work to AI may be driven by perceived benefits beyond cognitive ones, such as the belief it will result in a better grade or that it will take less time. Or, as pointed out in the section on employer expectations, graduates may be expected to interact with such systems as part of their employment. Regardless of why a student or university graduate might use an AI system, the key point that we can take from the current discussion is that research suggests we may overestimate the benefit of using such a system, for example believing we have learned more than they have actually learned, while failing to understand the risks, such as being more susceptible to false information or inappropriately confident in their own skills. Any curriculum we design must include mechanisms for ameliorating these effects by encouraging the development of metacognition.

## Metacognitive development

Development of metacognition emerges as a theme from the analysis of employer expectations for university graduates, cognitive frameworks of student learning, and research in cognitive science on the effect of “offloading” knowledge to technology. In each area, there is a reason that students must develop an awareness of their own cognitive processes and the ability to use that awareness to make better decisions about how to use technology appropriately. To improve the utility of AI in the curriculum instruction should focus on practices that deliberately promote building metacognitive skills. The purpose of this section is to provide a brief review of metacognition, with an emphasis on learning and technology in the classroom, in support of pedagogical recommendations for AI in the classroom.

Models and theories of metacognition differentiate between two distinct dimensions, (a) knowledge of cognition and (b) regulation of cognition (Flavell, 1979; Pintrich, 2002; Zohar & Barzilai, 2013). Metacognitive knowledge includes any knowledge that promotes one’s understanding of cognitive processing involved in completing a task (Pintrich, 2002; Rhodes, 2019). Based on Flavell’s (1979) classic article on metacognition, metacognitive knowledge can further be divided into three subcategories: strategic knowledge, knowledge about cognitive tasks, and self-knowledge. Strategic knowledge refers to knowledge of strategies for learning, thinking, and problem solving. Knowledge of cognitive tasks encompasses understanding how aspects of task conditions, demands, and goals influence cognitive activity. That is, knowledge that different tasks vary in difficulty and in turn, may require different cognitive strategies. Finally, self-knowledge describes knowledge of variables that influence individual cognitive activity, such as one’s strengths and weaknesses. Self-knowledge may also be referred to as knowledge of persons, or the knowledge of others’ cognitions (Zohar & Barzilai, 2013).

Metacognitive skills and metacognitive components are predominantly discussed when describing the self-regulation involved in metacognition (Chen & McDunn, 2022). Metacognitive skills are the skills and processes needed to monitor, control, and regulate cognition and learning, such as planning and evaluating (Whitebread et al., 2009). These skills further enhance students’ ability to evaluate the effectiveness of current strategies and progression toward goals as well as to organize one’s behavior during the learning process (Pintrich, 2002). Furthermore, metacognitive experiences, which have received less attention in research, describe the cognitive and affective experiences that arise when completing a cognitive task (Flavell, 1979; Zohar & Barzilai, 2013). An example of this could be the confidence a student feels that the answer they provided on an exam was correct (Rhodes, 2019). Whether conscious or non-conscious, these experiences are believed to be fundamental to the development and application of metacognitive skills (Chen & McDunn, 2022).

Metacognition plays a significant role in how students learn and perform in the classroom. Students who are aware of different strategies for learning, problem solving, and thinking are more likely to use them when attempting to recall information, study, or confront difficult classroom tasks (Pintrich, 2002). Metacognitive knowledge of these different strategies is believed to be transferable across settings (Rhodes, 2019). As computer-based learning environments which utilize AI both to provide structured and complementary feedback (as discussed with the Khan Academy work) and to provide support for knowledge acquisition and communication (such as when utilizing tools like ChatGPT) become more prevalent, students and faculty will be challenged to learn how to use them effectively to enhance classroom learning. Many researchers believe that new technologies can be used as metacognitive tools to foster learning about conceptually rich domains (Azevedo, 2005; Derry & Lajoie, 1993; Greene & Azevedo, 2010).

Azevedo (2005) describes several ways in which computers can act as a supportive tool for metacognition. This includes sharing cognitive load by supporting lower-level skills so that students can focus on higher-level thinking skills, providing learning opportunities that would otherwise not be accessible (e.g., electronic troubleshooting and just-in-time feedback). Therefore, any computer-enhanced learning environment that requires students to make decisions based on instructional goals, encourages decision making based on context, and promotes self-regulatory processes can be seen as a metacognition training tool (Azevedo, 2005; Gurbin, 2015). Further, instances in which computer environments fail to foster learning can often be attributed to students’ failure to use the metacognitive skills needed to regulate their learning in these new environments (Greene & Azevedo, 2010). But computer-enhanced learning must also be done within the context of what we understand about offloading cognition. In particular, using computer or AI components in the curriculum without educating students on possible cognitive risks such as misinformation, false confidence, and failures to learn may do more damage than good.

## Pedagogical recommendations for the AI age

Prioritizing metacognition particularly with respect to the use of tools that promote offloading, encouraging self-awareness and cognitive regulation, and supporting collaborative learning approaches that promote effective teamwork and technological integration, including AI, need to be pedagogical goals that are central in higher education instruction (Johnson & Johnson, 2008; Krathwohl, 2002). To maximize the benefits of collaborative learning (between fully human teams and between teams that include AI), pedagogical strategies must intentionally incorporate primary and secondary factors that encourage shared responsibility, interaction, and metacognitive skill development, enhancing student engagement and learning outcomes (Scager et al., 2016). But instructors must be mindful of the risks that incorporating technology into their curriculum and ensure students understand the risks. Below are some specific recommendations for practices to meet these goals across a variety of curricula in higher education.

## Make explicit the costs and benefits of using AI

A key theme from the literature on offloading cognition is that later, unaided, cognitive performance is worse. These costs can include an impoverished memory for information, inappropriate confidence in knowledge and skill development, and vulnerability to misinformation. The literature also demonstrates a lack of awareness of these effects on the part of the person that engaged in an offloading strategy. A lack of awareness is not special to the phenomenon of offloading. The psychology of learning literature has long recognized that learners are often unaware or wrongly aware of their own knowledge. (See resources such as Pashler et al., 2007, and Hacker et al., 2009, for more generalized recommendations to improve student learning despite metacognitive shortcomings.)

We should assume students will choose to offload some portion of their academic tasks, or we may include some form of offloading as part of assignments, so it is important to include a discussion of these risks. This can be as simple as including a lecture summarizing key findings such as those included here or a more active learning approach such as asking students to research and write on the topic or on specific subtopics such as misinformation susceptibility. Beyond lectures, assessments can serve as powerful tools to help students develop an accurate understanding of their own knowledge. Among the “strong” recommendations from the Pashler et al. (2007) report is the use of tests and quizzes to “re-expose students to key course content” (page 19) and help students self-assess their own learning. Using this practice is particularly important when learning might be impoverished due to cognitive offloading, especially when an instructor explicitly connects the practice to the development of better metacognitive performance and improved self-regulated learning (see Winne & Nesbit, 2009 for further discussion).

## Focus on higher-order outcomes and include AI as a tool

The original and revised versions of Bloom’s Taxonomy are structured to present learning outcomes that range from simple (knowledge or remember) to cognitively complex (evaluate or create). The revised version (Anderson & Krathwohl, 2001; Krathwohl, 2002) also includes a range of knowledge from factual knowledge to metacognitive knowledge. Lower-order outcomes can be more easily achieved by offloading cognition. For example, researching and summarizing facts is susceptible to the use of search engines to find readily available summaries accessible online. More sophisticated tasks such as analyzing and evaluating might be less susceptible to offloading to Google, but AI such as ChatGPT can be given information and asked to analyze and evaluate it. These tools can produce work that is unique, indistinguishable from good student work, and not readily detectable by plagiarism tools.

Higher-order knowledge in the revised taxonomy includes developing procedural and metacognitive knowledge as advanced outcomes. Assignments should include AI as part of the procedure of knowledge acquisition and evaluation and promote metacognition by requiring students to explicitly evaluate work from the AI system. For example, if a student is assigned the task of submitting a paper researching and evaluating a topic, the instructor could ask the student to first do the task using a tool like ChatGPT. The assignment could include the result as well as discussion of the prompt used to produce the work and outcomes when different prompts are used. The student might be asked to produce their own work after evaluating work produced by the tool by using other sources such as research and reading of original source materials. Students could reflect on the how their final submission developed as a process of work with “AI,” how accurate the products of AI were, explain the role of prompts in their results, reflect upon how they might use AI in future work and discuss how their knowledge of the topic was influenced by the AI and by other sources.

## More collaborative learning and include AI as a collaborator

Current methods of lecturing and prompting responses occasionally are not the best way to facilitate learning and engagement (Rau & Heyl, 1990). This keeps the instructor at the center of the classroom and takes the responsibility from the student. Collaborative learning groups place responsibility on students and make them accountable for their success. Group learning is a way to encourage students to participate in their education (Gunderson & Moore, 2008). It is also compatible with employer expectations of graduates, as covered in a previous section. While instructors may understand and prefer group learning, students may be uncertain about its use and avoid the idea due to fears of social loafing from their peers (Scager et al., 2016). To avoid this, instructors must consider both primary and secondary factors of the groups they are trying to implement.

Primary factors refer to design characteristics of the group, including the group size, group structure, task type. The most effective groups are small groups of three to five structured with students of mixed ability. This allows high achieving students to solidify the knowledge they have acquired, and low achieving students to learn the knowledge from their peers in a way that may be more digestible (Gunderson & Moore, 2008). More open-ended tasks facilitate deeper levels of reasoning that require interaction between the students (Scager et al., 2016). The secondary factors refer to characteristics of the group process or how instructors facilitate true and organic collaboration. Students must see everyone as a contributor to the success of the group to keep all members of the group accountable. The success of the team where everyone has a task or responsibility that the group will not succeed without should determine the outcome of the collaboration.

The advent of AI creates new possibilities. Tools like ChatGPT can become secondary collaborators in the student’s team. This is a form of computer-supported collaborative learning (CSCL) can improve group learning (Johnson & Johnson, 2008; Roberts, 2003) by providing new methods for communication and support for more productive student interactions (Roberts, 2003; Stahl et al., 2006). For example, AI systems can provide feedback or offer alternative views based on the information provided (Lo, 2023; Stahl, 2006). An AI system might be used as a neutral evaluator of group member contributions to allow students access to an unbiased assessment of their contribution to the group. Explicitly asking groups to include, make visible, and evaluate AI contributions should be part of any group assignment.

As AI becomes a more common part of work practice, employers will undoubtedly expect university graduates to be skilled in “AI as collaborator.” There remains an unanswered theoretical question of how humans will develop a “theory of mind” for these new systems and how that will impact their presence on a team. But instructors need not wait for theory to put incorporation of ChatGPT or similar tools on a team project into practice. Again, any such addition to the curriculum needs to be accompanied by task instructions making their use required, visible, and reflected upon so students can build critical metacognitive tools. As students engage in the practice of using the AI tools and justifying their reasoning for inclusion, or not, of the products of the tools, they will develop important skills that will generalize to any post-graduate endeavor. By embracing AI in the curriculum rather than trying to hold it at a distance, higher education will produce graduates better prepared for the new world they will find themselves in.

## Conclusion

Until recently, the advent of new technologies have enabled different means to “push” content and information to students during the learning process, but technologies have not replaced instructors in other critical ways. New AI tools may change that and require instructors in higher education to adopt approaches that build metacognitive knowledge, skills in metacognitive control, and skills of interpersonal and technological collaboration. Our fundamental recommendation is that higher education professionals can best serve learning outcome goals and the larger mission of education to provide a well-skilled workforce by embracing AI. There are clearly aspects of these new technologies that will make our jobs as educators more challenging. Nonetheless, we encourage the field to be creative and to see these tools as collaborators. AI can be a collaborator both for us, as instructors and educators, but also these systems must be accepted as collaborators of our students. Further, as students enter a workplace, their ability to work on human/computer “teams” is a critical cognitive skillset.

## Data Availability

No data were used in the preparation of this manuscript.
